# An Evaluation of the Quality of Life and Emotional Status of Patients With Implantable Cardioverter Defibrillators at the Vilnius University Hospital, Lithuania

**DOI:** 10.7759/cureus.68048

**Published:** 2024-08-28

**Authors:** Laura Nedzinskiene, Aldona Mikaliukstiene, Agne Kirkliauskiene, Agne Jakavonyte-Akstiniene, Vitalij Cernel, Ugne Utkute

**Affiliations:** 1 Department of Anatomy, Histology and Anthropology, Vilnius University, Vilnius, LTU; 2 Faculty of Medicine, Department of Nursing, Vilnius University, Vilnius, LTU; 3 Faculty of Medicine, Institute of Biomedical Sciences, Vilnius University, Vilnius, LTU; 4 Faculty of Medicine, Vilnius University, Vilnius, LTU

**Keywords:** sf-36, depression, qol, icd, heart rhythm disorders

## Abstract

Background and objective

An arrhythmia is a disorder of the heart rate or rhythm. An implantable cardioverter defibrillator (ICD) is a small electronic device connected to the heart to continuously keep track of and help control rapid and sometimes life-threatening electrical problems with the heart. However, it may result in psychological tension in patients’ lives, eventually affecting their quality of life (QoL). In light of this, we aimed to assess the QoL of patients with ICD at the Vilnius University Hospital, Lithuania.

Methods

We employed the following three questionnaires in this study: an originally prepared questionnaire including sociodemographic and health factors; the 36-item Short Form Health Survey questionnaire (SF-36); and the Hospital Anxiety and Depression Scale (HADS) to assess depression and anxiety. Data analysis was performed using SPSS Statistics v. 13.00 (IBM Corp., Armonk, NY).

Results

Of the patients evaluated in all areas of QoL, males demonstrated higher scores compared to females. A statistically significant difference was found when assessing the domains of physical activity, social function, pain, and physical and mental health QoL.

Conclusions

Based on our findings, the majority of ICD patients can achieve their desired QoL and psychosocial outcomes. Our results show that patients with ICDs have the potential to return to a normal life. Depression and anxiety manifested more commonly in respondents with a disease duration of up to five years.

## Introduction

An implantable cardioverter defibrillator (ICD) is a small electronic device connected to the heart. It is used to continuously keep track of and help control rapid and sometimes life-threatening electrical problems with the heart. ICDs have shown successful results in lowering mortality in patients with heart arrhythmias and those at high risk of sudden cardiac death (SCD) [1]. It effectively reduces death from ventricular arrhythmias in select patients, the risk of inappropriate shocks, and mortality from frequent therapy. The Antiarrhythmic Versus Implanted Defibrillators (AVID) trial showed that ICDs were more successful than antiarrhythmics in patients resuscitated from near-fatal ventricular fibrillation, or those with symptomatic prolonged ventricular tachycardia and hemodynamic compromise. In the Sudden Cardiac Death in Heart Failure Trial (SCD-HeFT), ICDs were also beneficial in lowering mortality in patients who were at elevated risk for SCD due to heart failure (HF) but did not have confirmed ventricular dysrhythmias. The benefits of ICDs can be fully realized only when the patient's quality of life (QoL) and psychological state are maintained [2].

Research suggests ICDs are more effective than pharmacological therapy in preventing SCD [3]. Although ICDs have shown lower mortality, their QoL and psychological effects are ambiguous and, as determined by the New York Heart Association (NYHA), vary depending on the functional class and age of the patient [4]. In six of seven studies, patients with ICDs had similar QoL to patients without ICDs. In one study, patients with ICDs had superior QoL than those who received pharmaceutical therapy [5]. After ICD implantation, almost a quarter of the patients experienced anxiety (25.3%) and depression (26 %). Regarding QoL, patients rated social life, energy or fatigue, emotional well-being, and pain moderately, or very poorly, and physical functioning poorly [6]. ICD implantation can be challenging, with early and late complications in the postoperative period, and patients have to be aware of the difficulties they might face for the rest of their lives [7]. This impacts patients' physical, mental, and social lives.

In cross-sectional studies, age was linked to poor QoL in patients with ICDs, and physical QoL improved in younger patients, but not in older patients throughout the first year following implant. There is no consensus on the link between the duration of ICD installation and QoL. In the populations studied, there is considerable variation in the length of follow-up, measurement of QoL, and degree of illness [1]. ICD recipients with anxiety and depression are at increased risk of mortality and hospital readmission [8]; therefore, evaluation of the risk factors for these psychological responses is key. Research has demonstrated the prevalence of depressive symptoms and the association with QoL in a large cohort of patients with HF and an implanted ICD. Of note, 43.2% of patients with HF and an ICD showed mild to severe depressive symptoms. In 16.8% of patients, depressive symptoms were moderate to severe. Depressive symptoms in patients with HF and an ICD were associated with low QoL [9].

In this study, we aimed to assess the QoL of patients with ICDs and to analyze various changes in people’s lives after the ICD implantation. We present the following two hypotheses in this study: (1) quality of life is poorer in older people with implanted ICD, and 2) anxiety and depression have a negative impact on QoL in patients with implanted ICD.

## Materials and methods

The study was carried out in the Cardiac Arrhythmia Department of Santaros Clinics of Vilnius University Hospital. The inclusion criteria were as follows: 1. adult male or female of various ages with implanted ICD; 2. duly completed questionnaire; 3. signed consent form for participation in the study; and 4. hospitalized in the Cardiac Arrhythmia Department of Santaros Clinics of Vilnius University Hospital. The patients were aided in the completion of questionnaires by a dedicated nurse, who had experience in such surveys.

Three questionnaires were used to conduct the study: 1. an originally prepared questionnaire, which consisted of questions about the gender, age, social group, marital status, and harmful habits (smoking) of the subjects. Also, the subjects were asked questions about the changes regarding their illness, duration of implantation of the ICD, changes in health status, QoL, physical activity, desire to participate in public life, work after implantation of the ICD; subjects were asked if they had other diseases and were asked to list them; 2. the 36-item Short Form Health Survey questionnaire (SF-36) [10]; and 3. the Hospital Anxiety and Depression (HAD) scale [11].

For the assessment of physical health (PH), the following parameters were employed: 1. physical activity (PA) - a person's ability to perform daily physical activity; 2. restriction of activity due to physical problems (PAR - physical activity restrictions); 3. pain (P): its duration, intensity, influence on daily activities; and 4. General Health Assessment (GHA), which shows how the patient assesses their own health.

For the assessment of mental health (MH), the following metrics were utilized: 1. vigor/vitality (VV), which shows the level of energy, freshness, and fatigue; 2. social function (SF), disclosing how health and emotional problems affect communication with relatives and friends; 3. activity limitation due to emotional problems and is influence on work and other activities (EAR - emotional activity restrictions); 4) emotional state (ES), which indicates various psychological states, especially anxiety and depression. Questions were used to assess each area, and the answers were evaluated using a point system, the sum of which can vary from 0 to 100 points. The higher the score, the better the QoL.

A total of 190 questionnaires were distributed. Out of the returned 190 questionnaires, 176 (92.6%) questionnaires were deemed suitable for the study.

Statistical analysis

SPSS Statistics v. 13.00 (IBM Corp., Armonk, NY), Excel 2003, and WinPEPI (v. 1.55) were used for data management and analysis. Means and medians were calculated to describe the general characteristics of continuous data. The frequency of categorical data was assessed in absolute numbers and percentages, confidence intervals (CI) were calculated, and ANOVA (analysis of variance) was used to compare averages. The chi-square (χ2) criterion was used to compare the frequencies of two or more variables - it determines the relationship between two or more nominal variables. The relationship between traits was considered statistically significant when p<0.05. An ANOVA test was used to compare independent samples. Differences between comparison groups were considered significant when p<0.05.

## Results

A total of 176 patients with ICD participated in the study: 48.9% (n=86) were male and 51.1% (n=90) were female; the mean age of the respondents was 66.98 ± 12,253 years. The participants were classified into various age groups as follows: up to 50 years, 50-59, 60-69, 70-79, and 80 years and over; 39.2% (n=69) of respondents were 60-69 years old and 28.4% (n=50) were in the age group of 70-79 years. Patients were categorized into groups according to the duration of ICD implantation: up to one year; one to three years; three to six years; six to nine years; and nine years and above (Table 1).

**Table 1:** Sociodemographic characteristics of participants ICD: implantable cardioverter defibrillator

Variables		N (%)
Sex	Male	86 (48.9)
	Female	90 (51.1)
Mean age, years: 66.98		
Age groups, years	Up to 50	14 (8)
	50–59	17 (9.6)
	60–69	69 (39.2)
	70–79	50 (28.4)
	80 and over	26 (14.8)
BMI, kg/m^2^,	18.5−24.9	35 (19.9)
	25−29.9	68 (38.6)
	30 and above	73 (41.5)
Duration of the implantation of the ICD, years	Up to 1	23 (13.1)
	1-3	47 (26.7)
	3-6	59 (33.5)
	6-9	31 (17.6)
	9 and above	16 (9.1)

The duration of heart rhythm disorder among the subjects ranged from 1 to 38 years (mean: 8.1 ± 6.01 years). A third (33.5%, n=59) of the respondents had ICD implanted for three to six years, a quarter (26.7%, n=47) for one to three years, 13.1% (n=23) for up to one year, and 17.6% (n=31) for six to nine years. The average duration of ICD implantation was 48.53 ± 35.112 months.

Compared to females, males had higher rates in all areas of QoL. A statistically significant difference (p<0.05) was found in the assessment of QoL domains: physical activity (PA), social function (SF), vigor/vitality (VV), pain (P), physical health (PH), and mental health (MH). Of all areas of QoL, both men and women rated overall health the lowest. Men evaluated physical and mental health with higher scores compared to women; 32.6% (n=28) of male respondents admitted to smoking. Compared to non-smokers, they had statistically significantly higher scores in the areas of QoL including physical activity, activity limitation due to physical problems, pain, and physical health (p<0.05). Most of the survey participants (89.8%, n=158) reported comorbidities. In addition to cardiac arrhythmia, 44.3% (n=78) had ischemic heart disease; 63.6% (n=112) had arterial hypertension (AH); 23.3% (n=41) had thyroid diseases; and 18.8% (n=33) had diabetes.

After analyzing the results of the assessment of QoL of the patients with heart rhythm disorders, taking into account the duration of the illness, it was observed that the duration of the illness had an impact on the assessment of all areas of the QoL (p<0.05), and it was statistically significant. Those with heart rhythm disorder had a worse assessment in all areas of QoL (Table 2).

**Table 2:** Assessment of respondents' QoL based on the duration of the heart rhythm disorder *P<0.05 QoL: quality of life; SF-36: 36-item Short Form Health Survey questionnaire; PA: physical activity; PAR: physical activity restrictions; EAR: emotional activity restrictions; SF: social function; ES: emotional state; VV: vigor/vitality; P: pain; GHA: general health assessment, PH: physical health; MH: mental health

SF–36 QoL areas	Disease duration, years				
	Up to 5	6−10	11−15	16 and above	
	Md ()	Md ()	Md ()	Md ()	
PA	65 (63.45)*	65 (57.78)	55 (51.36)	60 (56.00)	
PAR	75 (58.19)*	50 (52.78)	37.5 (38.64)	50 (41.67)	
EAR	100 (68.97)*	66.67 (56.79)	50 (48.49)	33.33 (44.44)	
SF	66.67 (65.13)*	66.67 (63.65)	61.12 (63.13)	55.56 (59.26)	
ES	68 (65.86)*	60 (59.11)	58 (59.09)	56 (59.47)	
VV	60 (56.12)*	55 (51.85)	52.5 (52.50)	50 (54.00)	
P	66.67 (67.82)*	55.56 (60.63)	66.67 (64.65)	55.56 (65.19)	
GHA	47 (44.67)*	45 (42.47)	42.5 (39.73)	35 (41.27)	
PH	49.5 (47.06)*	45 (44.62)	42 (42.58)	42 (43.99)	
MH	49.5 (46.62)*	43 (43.58)	42.5 (43.41)	44 (43.33)	

Further on the results of the evaluation of the areas of QoL, reviewing the duration of subjects after ICD implantation (Table 3) showed that a statistically significant number of individuals who had ICD implanted for up to one year have poorer QoL compared to respondents who had ICD implanted for one to three years (p<0.05). The results of the assessment of the QoL metrics of all groups (according to the duration of ICD implantation) showed that depending on the duration of ICD implantation, the assessment of the QoL worsened; a statistically significant difference was found in the following areas: PAR, SF, ES, VV, P, PH, and MH (p<0.05).

**Table 3:** Assessment of QoL in the entire study group based on the duration of ICD implantation *P<0.05: comparison of QoL areas in groups up to 11 months after ICD implantation and 12–35 months. **P<0.05: comparison of QoL areas in all ICD implantation groups QoL: quality of life; ICD: implantable cardioverter defibrillator; SF-36: 36-item Short Form Health Survey questionnaire; PA: physical activity; PAR: physical activity restrictions; EAR: emotional activity restrictions; SF: social function; ES: emotional state; VV: vigor/vitality; P: pain; GHA: general health assessment, PH: physical health; MH: mental health

SF–36 QoL areas	ICD implantation duration, years				
	Up to 1	1–3	3–6	6–9	9 and above
	Md ()	Md ()	Md ()	Md ()	Md ()
PA	45.00 (48.91)*	70.00 (64.04)	65.00 (58.90)	65.00 (60.00)	55.00 (53.75)
PAR	45.00 (31.52)*	75.00 (62.23)	50.00 (53.39)	50.00 (50.81)	50.00 (46.88)**
EAR	33.33 (43.48)*	100.00 (69.50)	66.67 (58.76)	66.67 (54.84)	66.67 (56.25)
SF	55.56 (57.01)*	77.78 (72.58)	55.56 (60.45)	66.67 (62.37)	61.12 (61.81)**
ES	56.00 (61.04)*	68.00 (68.94)	56.00 (57.08)	60.00 (60.90)	58.00 (56.25)**
VV	50.00 (50.43)*	70.00 (61.60)	55.00 (51.10)	50.00 (51.94)	45.00 (46.25)**
P	55.56 (57.97)*	66.67 (72.58)	66.67 (62.52)	55.56 (60.22)	55.56 (59.03)**
GHA	40.00 (39.26)*	50.00 (48.87)	45.00 (41.34)	45.00 (40.10)	42.50 (40.13)
PH	40.00 (41.11)*	48.00 (48.60)	47.00 (44.81)	45.00 (44.47)	42.00 (42.96)**
MH	41.00 (42.78)*	52.00 (49.17)	42.00 (42.69)	43.00 (43.87)	42.00 (41.56)**

Of note, 34.7% (n=61) of respondents reported to be in a depressive state. Average depression scores were higher in women (6.61 ± 4.04) compared to men (5.99 ± 4.04). In terms of a depressive state, statistically significant (p < 0.05) rates were seen more often in females (37.8%, n=34) compared to males (31.4%, n=27). A state of anxiety was reported in 44.3% (n=78) of all subjects. The average anxiety score was statistically significantly higher in women (8.01 ± 4.28) compared to men (6.56 ± 3.48) (p<0.015). Anxiety, as well as depression, was also more common (p<0.05) in women (48.9%, n=44) compared to men (39.5%, n=34).

Both anxiety and depressive states occurred less often in subjects who had a rhythm disorder for up to five years, compared to those who had it longer (p<0.05) (Table 4).

**Table 4:** Assessing depression and anxiety over the course of illness ^*^P<0.05

Duration of the disease, years	Number of subjects	Among the mentioned – depressive state	Among the mentioned - anxiety
		N (%)	N (%)
Up to 5	58	16 (27.6)*	20 (34.5)*
6−10	81	29 (35.8)	38 (46.9)
11−15	22	9 (40.9)	11 (50)
16 and above	15	7 (46.7)	9 (60)
		df=3; χ2=1.280; p=0.734	df=3; χ2=1.652; p=0.648

The frequency of depressive and anxiety states was evaluated according to the duration after ICD implantation. According to our study results, less than a year after ICD implantation, 43.3% (n=10) of the respondents were in a depressive state and 47.8% (n=11) in anxiety. The least manifestation of the depressive state and anxiety occurred in those subjects who were implanted with ICD for one to three years: depressive state: 21.3% (n=10); anxiety: 27.7% (n=13). After three to six years of ICD implantation, depression was found in 39% (n=23) and anxiety in 54.2% (n=32).

Our study results showed that statistically significant incidence of depressive state (22.2%, n=24) and anxiety (35.2%, n=38) occurred (p<0.05) less frequently in respondents after ICD implantation (Table 5).

**Table 5:** Evaluation of depressive state and anxiety as per the health assessment of all patients after ICD implantation *P=0.001l; **P=0.002 CI: confidence interval; ICD: implantable cardioverter defibrillator

Health status after ICD implantation	Number of subjects	Among the mentioned – depressive state		Among the mentioned - anxiety	
		%	95% CI	%	95% CI
Greatly improved	108	24 (22.2)*	14.78–30.49	38 (35.2)**	26.24–44.39
Slightly improved	55	35 (63.6)*	49.56–75.71	36 (65.5)**	51.42–77.32
Not improved	13	2 (16.7)	1.92–38.48	4 (33.3)	9.09–57.20
		χ2=13.29; p=0.001		χ2=6.38; p=0.041	

Depressive state and anxiety were found more often in respondents who indicated that their work was affected after ICD implantation as compared to those who indicated that they could work even more after ICD implantation (p<0.01). The dependence of the depressive state on the ability to work (χ2=8.342; p=0.039) after ICD implantation was determined.

We observed a statistically significant negative correlation between the evaluation of the QoL after implanting an ICD and the occurrence of depression (r=-0.215; p=0.004), i.e., as the assessment of the QoL improved, the occurrence of depression decreased. A statistically significant negative weak correlation was also observed between the evaluation of the QoL after ICD implantation and the manifestation of anxiety (r=-0.164; p=0.03), i.e., as the QoL assessment improved, anxiety decreased.

## Discussion

The concept of QoL has several dimensions. It is a foundational concept of health (physical, mental, and social) and encompasses all aspects of an individual's life. Health and human functional status are only two dimensions of QoL. Most research focusing on treatment results also examines QoL from the patient's point of view [11]. The published data about the influence of age on QoL assessment vary and is often contradictory. Some authors suggest that age is associated with fear and pessimism and deteriorating physical, social, and emotional health [12]. A person should not lose self-esteem until they are very old. Physical capacity and health are very important as they allow a person to remain independent and able to take care of themselves, and not depend on the direct help of other people; consequently, social relations are often affected. As people age, their physical, social, and economic power decreases, and hence social risks increase. All this affects the QoL - increasing the chances of developing QoL risks. Other authors have contrasted these findings, claiming that older age is not an obstacle for a person to be happy and enjoy life [13].

Several studies show that most elderly people rate their health and material and social situation fairly well. A study among elderly people living independently investigated the correlation between frailty and QoL. Many of them had relatively low overall frailty scores. The values related to QoL of the elderly, and general satisfaction with their health status were quite high [14]. Our findings showed that estimation of the QoL in the areas of physical activity, its limitation due to physical restrictions, pain, and physical health decreased with age. The QoL was rated highly by subjects under 50 and 50-59 years of age. Those aged 70 years and older rated all areas of QoL poorly.

Swedish researchers evaluated sociodemographic changes in QoL in patients with ICD and found that QoL was better in men, as well as in subjects who were married and had their own housing. The duration of ICD implantation also had an impact on QoL ratings. Women, the elderly, single people, and retirees had a lower QoL score [15]. In another study, statistically significant improvements were observed in general well-being, sleep, appetite, and physical activity (p<0.05), but not in cognitive function, involvement in social life, ability to work, and sexual function (p>0.05) [16].

According to our study findings, the duration of the illness had an impact on the assessment of all areas of QoL (p<0.05); the longer the duration of the illness, the worse the assessment of QoL. We assessed the changes in QoL depending on the disease and duration of ICD implantation. The results of the evaluation of the QoL areas, after reviewing the duration of ICD implantation in subjects, showed that respondents who had ICD implanted for up to one year evaluated all areas of QoL with lower scores compared to those who had ICD implanted for one to three years; the difference was statistically significant (p<0.05).

The results of the evaluation of QoL areas of all the groups (according to ICD implantation duration) showed a statistically significant difference in the following areas: PAR, SF, ES, VV, P, FS, and MH (p<0.05), indicating that up to one year after implantation and nine years and longer the person lives with ICD, the worse the scores of the QoL assessment. Besides heart rhythm disorder, 158 subjects also suffered from other diseases: ischemic heart disease, arterial hypertension, thyroid gland oncological diseases, and diabetes. The areas of QoL were rated statistically significantly (p<0.05) with higher scores among subjects who did not have other diseases. According to some research, patients' dependence on ICD can increase anxiety and limit their functioning in various areas of life, thereby worsening QoL. To improve the quality of care for such patients, it is necessary to thoroughly understand the psychological factors that determine QoL [17]. Patients with such medical conditions and with implanted ICDs are more emotionally vulnerable and have more difficulties in social adaptation, especially in older age.

In our research, we assessed the changes in depression and anxiety states by taking into account social (gender, age, marital status) and disease (duration of the disease and ICD implantation, other diseases) factors. Another study found that one year after implantation, patients had a better quality of life compared to before implantation, but had a lower overall health score four years after implantation [18]. Similarly, physical functioning was rated as good at one-year post-implantation and progressively worse thereafter. We also compared the frequencies of depression and anxiety, by reviewing patients' assessments of health, QoL, well-being, physical activity, desire to participate in social life, and ability to work after ICD implantation.

In our study, depressive state was seen in 34.7% (n=61) and anxiety in 44.3% (n=78) of all respondents. Depressive state and anxiety occurred more often in women (37.8%, n=34) compared to men (31.4%, n=27). Depression and anxiety were more common in respondents aged 70 years and older. Less than a year after ICD implantation, 43.3% (n=10) of the respondents were in a depressive state, while 47.8% (n=11) had anxiety. The lowest manifestation rate of depressive state and anxiety appeared in the subjects who had been implanted with ICD for one to three years. While it is clear that ICD implantation is important for improving QoL, comorbidities in the elderly do not allow a correct assessment. The rate of depression depends on the presence of comorbidities; health assessment; changes in physical activity; and the possibility of working after the implantation of an ICD. Anxiety depends on health assessment and the changes in physical activity after the implantation of an ICD.

Our study has a few limitations, which could be addressed in future research. Primarily, we had a relatively smaller sample size. Secondly, our study design was not fully suitable for a study of this nature, and hence we recommend case-control studies to investigate and account for every possible lifestyle factor and their impact on this population.

## Conclusions

The majority of ICD patients can achieve their desired QoL goals and psychosocial outcomes. Our results and those of other studies show that patients with ICDs have the potential to return to a normal life; however, they and their physicians need to continuously monitor their health status. These psychosocial issues are just a few examples of the common contemporary reality of living successfully with heart disease. Researchers and clinicians continue to make progress to effectively plan and manage these critical events and achieve the desired patient QoL outcomes.

Physical activity, social functioning, energy/vitality, pain, and physical and mental health are the areas of QoL rated better by men (p<0.05). The longer the duration of ICD implantation, the worse the assessment of QoL in areas of restricted activity related to physical problems, social functions, energy/vitality, pain, and physical and mental health. Depression and anxiety manifested more commonly in respondents with a disease duration of up to five years and had ICD implanted for one to three years. QoL in terms of physical functioning and comfort decreases with increasing age, and the longer the ICD is implanted, the worse the QoL in terms of functional vitality. Gender, age, and duration of implantation influence QoL assessment and should be considered while devising strategies to address QoL in patients with ICD implantation.

## Appendices

**Table 6:** 36-Item Short Form Survey Instrument (SF-36)

Choose one option for each questionnaire item												
1. In general, would you say your health is	1	Excellent	◯									
	2	Very good	◯									
	3	Good	◯									
	4	Fair	◯									
	5	Poor	◯									
2. Compared to one year ago, how would you rate your health in general now?												
	1	Much better now than one year ago				◯						
	2	Somewhat better now than one year ago				◯						
	3	About the same				◯						
	4	Somewhat worse now than one year ago				◯						
	5	Much worse now than one year ago				◯						
The following items are about activities you might do during a typical day. Does your health now limit you in these activities? If so, how much?												
	Yes, limited a lot		Yes, limited a little		No, not limited at all							
3. Vigorous activities, such as running, lifting heavy objects, participating in strenuous sports	1	◯	2	◯	3	◯						
4. Moderate activities, such as moving a table, pushing a vacuum cleaner, bowling, or playing golf	1	◯	2	◯	3	◯						
5. Lifting or carrying groceries	1	◯	2	◯	3	◯						
6. Climbing several flights of stairs	1	◯	2	◯	3	◯						
7. Climbing one flight of stairs	1	◯	2	◯	3	◯						
8. Bending, kneeling, or stooping	1	◯	2	◯	3	◯						
9. Walking more than a mile	1	◯	2	◯	3	◯						
10. Walking several blocks	1	◯	2	◯	3	◯						
11. Walking one block	1	◯	2	◯	3	◯						
12. Bathing or dressing yourself	1	◯	2	◯	3	◯						
During the past 4 weeks, have you had any of the following problems with your work or other regular daily activities as a result of your physical health?												
	Yes		No									
13. Cut down the amount of time you spend on work or other activities	1	◯	2	◯								
14. Accomplished less than you would like	1	◯	2	◯								
15. Were limited in the kind of work or other activities	1	◯	2	◯								
16. Had difficulty performing the work or other activities (for example, it took extra effort)	1	◯	2	◯								
During the past 4 weeks, have you had any of the following problems with your work or other regular daily activities as a result of any emotional problems (such as feeling depressed or anxious)?												
	Yes		No									
17. Cut down the amount of time you spend on work or other activities	1	◯	2	◯								
18. Accomplished less than you would like	1	◯	2	◯								
19. Didn't do work or other activities as carefully as usual	1	◯	2	◯								
20. During the past 4 weeks, to what extent have your physical health or emotional problems interfered with your normal social activities with family, friends, neighbors, or groups?	1	◯	Not at all									
	2	◯	Slightly									
	3	◯	Moderately									
	4	◯	Quite a bit									
	5	◯	Extremely									
21. How much bodily pain have you had during the past 4 weeks?	1	◯	None									
	2	◯	Very mild									
	3	◯	Mild									
	4	◯	Moderately									
	5	◯	Severe									
	6	◯	Very severe									
22. During the past 4 weeks, how much did pain interfere with your normal work (including both work outside the home and housework)?	1	◯	Not at all									
	2	◯	A little bit									
	3	◯	Moderately									
	4	◯	Quite a bit									
	5	◯	Extremely									
These questions are about how you feel and how things have been with you during the past 4 weeks. For each question, please give the one answer that comes closest to the way you have been feeling. How much of the time during the past 4 weeks…												
	All of the time		Most of the time		A good bit of the time		Some of the time		A little of the time		None of the time	
23. Did you feel full of pep?	1	◯	2	◯	3	◯	4	◯	5	◯	6	◯
24. Have you been a very nervous person?	1	◯	2	◯	3	◯	4	◯	5	◯	6	◯
25. Have you felt so down in the dumps that nothing could cheer you up?	1	◯	2	◯	3	◯	4	◯	5	◯	6	◯
26. Have you felt calm and peaceful?	1	◯	2	◯	3	◯	4	◯	5	◯	6	◯
27. Did you have a lot of energy?	1	◯	2	◯	3	◯	4	◯	5	◯	6	◯
28. Have you felt downhearted and blue?	1	◯	2	◯	3	◯	4	◯	5	◯	6	◯
29. Did you feel worn out?	1	◯	2	◯	3	◯	4	◯	5	◯	6	◯
30. Have you been a happy person?	1	◯	2	◯	3	◯	4	◯	5	◯	6	◯
31. Did you feel tired?	1	◯	2	◯	3	◯	4	◯	5	◯	6	◯
32. During the past 4 weeks, how much of the time have your physical health or emotional problems interfered with your social activities (like visiting with friends, relatives, etc.)?	1	◯	All of the time									
	2	◯	Most of the time									
	3	◯	Some of the time									
	4	◯	A little of the time									
	5	◯	None of the time									
How TRUE or FALSE is each of the following statements for you?												
	Definitely true		Mostly true		Don't know		Mostly false		Definitely false			
33. I seem to get sick a little easier than other people	1	◯	2	◯	3	◯	4	◯	5	◯		
34. I am as healthy as anybody I know	1	◯	2	◯	3	◯	4	◯	5	◯		
35. I expect my health to get worse	1	◯	2	◯	3	◯	4	◯	5	◯		
36. My health is excellent	1	◯	2	◯	3	◯	4	◯	5	◯		

**Table 7:** Hospital Anxiety and Depression Scale (HADS) Please check you have answered all the questions Scoring: Total score: depression (D): Anxiety (A) [0-7=normal; 8-10=borderline abnormal (borderline case); 11-21: abnormal (case)]

D	A		D	A	
		I feel tense or 'wound up':			I feel as if I am slowed down:
	3	Most of the time	3		Nearly all the time
	2	A lot of the time	2		Very often
	1	From time to time, occasionally	1		Sometimes
	0	Not at all	0		Not at all
		I still enjoy the things I used to enjoy:			I get a sort of frightened feeling like 'butterflies' in the stomach:
0		Definitely as much		0	Not at all
1		Not quite so much		1	Occasionally
2		Only a little		2	Quite Often
3		Hardly at all		3	Very Often
		I get a sort of frightened feeling as if something awful is about to happen:			I have lost interest in my appearance:
	3	Very definitely and quite badly	3		Definitely
	2	Yes, but not too badly	2		I don't take as much care as I should
	1	A little, but it doesn't worry me	1		I may not take quite as much care
	0	Not at all	0		I take just as much care as ever
		I can laugh and see the funny side of things:			I feel restless as I have to be on the move:
0		As much as I always could		3	Very much indeed
1		Not quite so much now		2	Quite a lot
2		Definitely not so much now		1	Not very much
3		Not at all		0	Not at all
		Worrying thoughts go through my mind:			I look forward with enjoyment to things:
	3	A great deal of the time	0		As much as I ever did
	2	A lot of the time	1		Rather less than I used to
	1	From time to time, but not too often	2		Definitely less than I used to
	0	Only occasionally	3		Hardly at all
		I feel cheerful:			I get sudden feelings of panic:
3		Not at all		3	Very often indeed
2		Not often		2	Quite often
1		Sometimes		1	Not very often
0		Most of the time		0	Not at all
		I can sit at ease and feel relaxed:			I can enjoy a good book or radio or TV program:
	0	Definitely	0		Often
	1	Usually	1		Sometimes
	2	Not Often	2		Not often
	3	Not at all	3		Very seldom
